# Supplementary material for: “After a long day of play, I get tired and forget to unfurl my bed net”: a qualitative study exploring factors affecting insecticide-treated bed net use among school-aged children in eastern Uganda
Source: Malar J. 2025 Nov 28;25:1. doi: 10.1186/s12936-025-05653-7 (PMC12763868; doi:10.1186/s12936-025-05653-7)
Supplement: Supplementary file 1 — Supplementary Material 1 [file 12936_2025_5653_MOESM1_ESM.docx]

**Appendix B:**

**Qualitative Interview Guide**

*Now I am going to ask you some detailed questions. I have a series of questions that I will ask you to guide our conversation. Please feel free to stop me at any point during our conversation to tell me your thoughts about other things that you think are important to this topic, even if I don’t ask specifically about them. There are no right or wrong answers. Please share as much information as you feel comfortable sharing.*

1. First, I would like to know about your own thoughts in general about bednet use:
   1. In general, what do you think about the bednets themselves?
      1. *Probe: What about them do you feel is positive or negative?*
   2. What do you feel like are the benefits of bednet use?
      1. *Probe: Do you feel like they help prevent malaria for yourself?*
      2. *Probe: Do you feel like there are other benefits you have experienced from using a bednet?*
   3. Do you feel using a bednet is harmful in any way?
      1. *Probe: If so, what risks do you feel there are to using a bednet?*
      2. *Probe: Do you dislike sleeping under the net? If so, why?*
   4. How do you feel about using a bednet nightly?
      1. *Probe: Does it feel effective to use it every night or do you prefer to use it differently?*
      2. *Probe: Do you worry when you don’t use a bednet every night? How does it make you feel to not use a bednet nightly?*
      3. *Probe: Do you have other uses for your bednet? If so, what are they?*
   5. When you do use a bednet, why do you choose to use it? What makes using a bednet easier for you?
   6. When you want to use a bednet but don’t, what do you think stops you from doing it? What makes using a bednet difficult for you?
   7. What aspects of your identity may affect your choice to use a bednet?
      1. *Probe topics: Is it your age? What village or tribe you are from? If you are pregnant? If you are female? If you have other illnesses?*
      2. Follow-up: Why do feel these factors affect the way you use bednets?
2. Next, let’s talk about bednet use in your household and community
   1. How do you think members of your community expect people to use bednets?
      1. *Probe: What expectations are there within your own household that members of the household should use bednets?*
      2. *Probe: What about expectations within your larger community?*
      3. *Do these expectations affect how you feel about using a bednet?*
   2. Do all members of your household sleep under a bednet? Why or why not?
      1. Follow-up: Is this a choice you make as a group or individually? How does that affect how you feel about bednets?
   3. How do you feel when members of your household choose to use a bednet differently from you?
      1. *Probe: Do you feel your bednet use is affected by those you live with? Why do you feel it matters?*
   4. Do you feel your household’s sleeping arrangement can change the way you use a bednet?
      1. *Probe: What about the sleeping arrangement can make using bednets easier or more challenging? Does it matter the number of rooms? How about the number of nets?*
      2. *Probe: What would be the ideal sleeping arrangement for everyone in your household to be able to use a bednet?*
   5. What aspects of your household may make you want to use a bednet more?
      1. *Probe: If someone in your household was pregnant? If there are children <5 years old? If there is someone who is already sick?*
      2. *Probe: Does the number of people in your home change how you use bednets?*
   6. Do other people in your family use bednets differently from yourself?
      1. *Probe: If you’re a parent, do your kids use it differently? Or if you’re a child, do your parents use it differently?*
      2. *Probe: Why do you think different family members might use bednets differently? How does that make you feel?*
   7. Do you feel using your own bednet also affects your household’s chance of getting malaria? Why or why not? How about the community’s chance of getting malaria?
3. Next, let’s talk about your understanding of bednets and malaria
   1. What is your understanding of malaria and how malaria is transmitted?
      1. *Probe: How did you learn about malaria? What resources did you have to learn about malaria?*
      2. Follow-up: Do you feel it would be helpful to learn more about malaria transmission?
   2. What is your understanding about how bednets prevent malaria?
      1. *Probe: How did you learn about bednets?*
      2. *Probe: What is your education level? Do you feel this changes the way you understand bednets and malaria?*
      3. *Probe: How does your understanding of bednets and malaria prevention affect whether you choose to use a bednet?*
      4. Follow-up: Do you feel it would be helpful to learn more about bednets and how they prevent malaria?
   3. Who do you feel should use bednets?
      1. P*robe: Does the age of the person affect how they should use a bednet? How about if the person is pregnant?*
   4. What do you feel makes a bednet most effective against malaria?
      1. *Probe: How would you use a bednet to prevent malaria?*
      2. Follow-up: If you wouldn’t use a bednet nightly to prevent against malaria, why not? Do you still feel like your method is effective for preventing malaria?
   5. Have you learned about other strategies of malaria prevention, such as Indoor Residual Spraying (IRS)?
      1. *Probe: If so, how do you feel about using IRS compared to bednets?*
      2. *Probe: Do you feel it’s beneficial to use both methods? If you have access to both IRS and bednets, which one do you prefer to use?*
4. Next, let’s talk about your access to bednets and the quality of nets
   1. Do you have a bednet currently or have in the past? If so, how did you access the net?
   2. How many nets do you have in your household? Do you feel this is enough? How does the number of nets in your household affect how you use them?
   3. When you have a bednet, how long do you usually use it for? What makes you stop using one?
   4. How easy or difficult is it to use a bednet? What about a bednet makes it this way?
      1. *Probe: Do you feel like this prevents you from using one?*
      2. *Probe: Would you like more teaching on how to use a bednet? Do you feel like this would be helpful?*
      3. *make them easier to use?*
5. Let’s also talk about your access to healthcare and experiences with malaria
   1. What do you do when you get malaria?
      1. *Probe: How do you feel about getting malaria? Does getting malaria concern you?*
      2. *Probe: Do you worry about getting malaria in the future? How much of a risk do you feel malaria poses to your health?*
   2. Have you had malaria before? If so, what was that like?
      1. *Probe: How do your previous experiences with malaria affect how you feel about preventing malaria? Do they affect how you feel about bednet use?*
   3. What are your experiences with other people you know who have had malaria?
      1. *Probe: Do these experiences affect how you feel about preventing malaria? Do they affect how you feel about bednet use?*
6. Next, let’s talk about how the environment impacts bednet use
   1. How easy or difficult does your sleeping arrangements make bednet use? What about your arrangements makes it this way?
   2. Do you have multiple places where you sleep? If so, how do you use bednets in the respective places?
      1. *Probe: What about the different places makes it easier or harder to use bednets?*
      2. Follow-up: If you had enough bednets, do you feel like you would use them in all the places you sleep?
      3. *Probe: How easy or difficult do you feel bednets are to travel with? Do you wish they were more portable?*
   3. Do you feel living in an urban or city environment would change how use bednets, compared to rural or village? Why is that?
      1. *Probe: How about using bednets in different rural villages or districts?*
      2. *Probe: Do you feel there is a different malaria risk depending on what environment you’re in? Does that change how you use bednets?*
   4. Do you have reasons to get up during the night, such as taking care of young kids?
      1. *Probe: Does this affect how you use a bednet? Do you notice yourself furling and unfurling them several times during the night? How does this make you feel?*
   5. Do you notice a lot of mosquitoes when you sleep?
      1. *Probe: Does this affect your decision to use a bednet? Do you feel a bednet impacts the number of mosquitoes you notice?*
   6. Does the time of year affect your bednet use?
      1. *Probe: For example, do times of rainy season change how you use bednets? Why? How about heat?*
7. Next, let’s talk about incorporating bednet use into your every-day life
   1. Do you feel financially secure? Is acquiring a bednet a stress to you financially?
      1. *Probe: Does that affect how bednets are used in your household?*
   2. Do you and your household have enough food to eat? Does this affect how you prioritize bednet use?
      1. *Probe: If your food situation were to change, would you anticipate a change in how you use a bednet?*
   3. Do you have a reliable place to sleep at night? Do you worry about being safe where you sleep?
      1. *Probe: How does this affect your bednet use?*
   4. Does your occupation make it more difficult to use a bednet? How so?
      1. *Probe: What do you do when you encounter occupation challenges that prevent you from using a bednet? How do you feel about these challenges?*
   5. How do you feel using a bednet fits into your routines? Is it easy or difficult?
      1. *Probe: What about your routines makes it easy or difficult to use a bednet?*
      2. *Probe: Have you ever adapted your routine to better use a bednet? How so? Why did you adapt?*
   6. How often do you feel like you forget to use a bednet when you intend to?
      1. *Probe: What makes it easier to remember? What makes it easier to forget? Have you found ways to help you remember?*
   7. How often do you not use a bednet due to fatigue or tiredness?
      1. *Probe: Have you used ways to counteract this, such as putting up the bednet earlier in the day? How do you feel about using a bednet when you’re very tired compared to when you have more energy?*
      2. *Probe: Do you feel there are resources to help overcome challenges in bednet use?*
      3. Follow-up: If not, would you like more resources on how to make a bednet adaptable for your lifestyle?
8. Lastly, let’s talk about a special type of bednet, SmartNet. This device is just like a regular bednet, but it measures how much you are using the bednet overnight.
   1. Would you be interested in using this bednet? Why or why not?
   2. What questions or concerns would you have about the bednet?
   3. What are other ways that you feel like would be most effective in helping you use a bednet more often?
